# Disparities in colorectal cancer screening among breast and prostate cancer survivors

**DOI:** 10.1002/cam4.3729

**Published:** 2021-02-05

**Authors:** Chiranjeev Dash, Jiachen Lu, Vicky Parikh, Stacey Wathen, Samay Shah, Ruchi Shah Chaudhari, Lucile Adams‐Campbell

**Affiliations:** ^1^ Office of Minority Health and Health Disparities Research Georgetown Lombardi Comprehensive Cancer Center Washington DC USA; ^2^ MedStar Shah Medical Group MedStar Health Washington DC USA

**Keywords:** breast cancer, cancer survivorship, colonoscopy, colorectal cancer, prostate cancer, screening

## Abstract

**Background:**

Colorectal cancer (CRC) screening is recommended as an integral part of cancer survivorship care. We compared the rates of CRC screening among breast and prostate cancer survivors by primary cancer type, patient, and geographic characteristics in a community‐based health‐care system with a mix of large and small metro urban areas.

**Materials and Methods:**

Data for this retrospective study were abstracted from medical records of a multi‐specialty practice serving about 250,000 individuals in southern Maryland. Breast (N = 1056) and prostate (N = 891) cancer patients diagnosed prior to 2015 were followed up till June 2018. Screening colonoscopy within the last 10 years was considered to be guideline concordant. Multivariate logistic regression was used to determine the prevalence odds ratios of being concordant on CRC screening by age, gender, race, metro area type, obesity, diabetes, and hypertension.

**Results:**

Overall 51% of survivors had undergone a screening colonoscopy. However, there was a difference in CRC screening rate between prostate (54%) and breast (44%) cancer survivors. Older age (≥65 years), being a breast cancer survivor compared to prostate cancer, and living in a large compared to small metropolitan area were associated with a lower probability of receiving CRC screening. Having hypertension was associated with higher likelihood of being current on colonoscopy screening guidelines among survivors; but diabetes and obesity were not associated with CRC screening.

**Conclusions:**

Low levels of CRC screening utilization were found among breast and prostate cancer survivors in a single center in Southern Maryland. Gender, comorbidities, and residential factors were associated with receipt of CRC screening.

## Graphical abstract

Colorectal cancer screening is recommended as part of survivorship care among breast and prostate cancer survivors. We examined the disparities in receipt of colonoscopy screening among breast and prostate cancer survivors in a community‐based health‐care system. Overall, we found low rates of colorectal cancer screening among survivors (51%). Older survivors, breast cancer as compared to prostate cancer survivors, and those living in large compared to smaller metropolitan areas were less likely to have received colonoscopy screening.

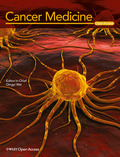

## 1 INTRODUCTION

Breast and prostate cancers are the most common cancers among women and men, respectively, in the United States. Improvements in cancer detection and treatment rates for these cancers have resulted in improved survival with 90% of breast and 99% of prostate cancer survivors expected to live beyond 5 years post‐diagnosis.^1^ However, cancer survivors have a greater risk of developing second primary cancers which has led to the IOM calling for a critical need to improve long‐term follow‐up of cancer survivors.^2^, ^3^, ^4^, ^5^, ^6^ This includes screening for common cancers, such as colorectal cancer (CRC), the most preventable and treatable cancer, and the third most common cause of cancer and cancer death in the United States.^1^

Several studies among breast cancer survivors have reported an increased risk of a second primary CRC compared to the general population.^6^ Similarly, a second primary CRC is also more likely among prostate cancer survivors compared to men without a personal history of cancer suggesting high‐risk profiles for CRC in survivors of these cancers.^7^ CRC screening, therefore, should be a high priority for the survivorship care after these cancers.

Evidence on whether cancer survivors are more likely to undergo screening compared to those without cancer is mixed. Several studies suggest that patients with cancer are more likely to receive mammograms, pap smears, and PSA tests than those without a history of cancer,^8^, ^9^ whereas others have either found no difference^10^ or a decreased likelihood of screening among cancer survivors.^11^ It is also unclear whether persistent racial/ethnic disparities in CRC screening seen in the general population is also evident among breast and prostate cancer survivors.^12^ Breast cancer survivors are more likely to receive CRC screening than the general population^13^ but evidence in prostate cancer survivors is limited. However, comparison of screening rates within the survivor population; and comparison between different types of cancers, such as breast and prostate, has not been reported.

Most of the research on cancer screening among survivors has focused on system‐level issues such as provider type and patient QOL.^14^, ^15^ More than 90% of cancer survivors report visiting their PCPs for their continuing care which makes physician recommendation for primary screening the biggest predictor of receiving screening.^16^ In studies with multiple provider types and physicians from different systems, the effect of factors other than physician recommendation is hard to measure. Studies from a single health system would help to address this issue.

Although previous studies have suggested that rural populations are less likely to receive CRC screening than those living in metropolitan urban areas,^17^, ^18^, ^19^, ^20^ it is not known whether there might be differences between small and large urban areas. Moreover, demographic, geographic, and medical history factors affecting cancer screening have not been investigated across different cancers. We compared the rates of CRC screening among breast and prostate cancer survivors using a single community‐based health‐care system in Southern Maryland––which has a mix of large and small metro urban areas.

## 2 MATERIALS AND METHODS

Data for the cross‐sectional study were abstracted from the EMR of MedStar Shah Medical Group. MedStar Shah Medical Group is a multi‐specialty (including oncology) practice that offers outpatient medical services in the Southern Maryland area. The practice includes 21 locations and serves the medical needs of an estimated population of 250,000 individuals across five counties in the state of Maryland. The study was approved by the Georgetown University‐MedStar IRB.

Medical records of breast and prostate cancer patients diagnosed prior to 2015 were abstracted, and CRC screening data through June 2018 was recorded. Participants eligible for the study had to be 50 years of age or older (in accordance with USPSTF CRC screening recommendations for asymptomatic average risk adults) as of 30 June 2018 and have no personal history of CRC.^21^ Data on age, gender, race/ethnicity, residential zip code, primary cancer type (breast/prostate), date of diagnosis, and current vital status were abstracted from the electronic medical records. Comorbidities, specifically, obesity, diabetes, and hypertension prevalence were also abstracted from the medical records. Zip codes and associated Rural Urban Continuum Codes (RUCC, 2013; Economic Research Service, United States Department of Agriculture) were used to determine the urban residence type and were categorized into the following categories for analyses––metro area ≥250,000 or metro area <250,000 population. The final study population included 1,947 cancer survivor patients (1,056 breast cancer survivors and 891 prostate cancer survivors).

Colorectal cancer screening status was determined using USPSTF guidelines.^21^ Patients screened within the last 10 years by having a colonoscopy performed was considered to be guideline concordant. Patients who were screened prior to their cancer diagnosis were considered adherent to guidelines if the last recorded screening was less than 10 years from 30 June 2018 for this cross‐sectional analysis. Colonoscopy was the primary method of screening recommended by physicians at MedStar Shah Medical Group. All CRC screening data were abstracted from medical and billing records using current procedural terminology (CPT) codes (45378 and ICD‐9/ICD‐10 code V76.51/Z12.11) or Healthcare Common Procedural Coding System (HCPCS) codes (G0105 and G0121) to identify the screening colonoscopy.

### 2.1 Statistical analysis

Cancer survivor demographic characteristics are reported as means (SD) for continuous, and frequency for categorical variables. Characteristics are reported for all survivors, and separately for breast and prostate cancer survivors. Multivariate logistic regression analyses were used to determine prevalence odds ratios of being concordant on CRC screening by age, gender, race, metro area type, obesity, diabetes, and hypertension. Separate logistic regression analyses were conducted for both survivor groups combined and individually for breast and prostate cancer groups. We also stratified the sample by metro area subtype to determine whether characteristics associated with CRC screening might differ among cancer survivors based on geography. Finally, given that screening recommendations do not recommend routine screening for all individuals above 75 years of age, we conducted additional analyses after excluding participants 75 years of age and above from our analytic dataset (Tables S1 and S2). All tests were two‐sided, and statistical significance was defined as a *p* value <0.05. All analyses were performed using SAS 9.3 (SAS Corp).

## 3 RESULTS

Baseline characteristics of the 1947 cancer survivors (1056 breast and 891 prostate) included in the study are presented in Table 1. At the time of data abstraction, the mean age was 71.22 years with 30% of the participants ≥75 years of age, and prostate cancer survivors being, on average, about 4 years older than the breast cancer survivors. Overall, 57% of the survivors self‐reported to be Non‐Hispanic White (NHW) and 30% Non‐Hispanic Black (NHB). Based on their zip code of residence, 58% of the survivors reported living in a large metro area (population ≥250,000) compared to 42% in smaller metro areas, that is, population <250,000. Mean BMI was 29.57 and the proportion of patients with hypertension and diabetes was 73% and 28%, respectively. (Table 1).

**TABLE 1:** Baseline characteristics of breast and prostate cancer survivors.

Characteristics	All Survivors (N = 1947)	Breast cancer Survivors (N = 1056)	Prostate cancer Survivors (N = 891)
Age (in years), mean (SD)	71.22 (10.35)	69.36 (10.56)	73.43 (9.65)
Age at diagnosis, mean (SD)	69.22 (10.33)	67.36 (10.57)	71.41 (9.59)
<50	49 (2.52)	44 (4.17)	5 (0.56)
50–64	634 (32.56)	407 (38.54)	227 (25.48)
65–74	680 (34.93)	340 (32.2)	340 (38.16)
≥75	584 (29.99)	265 (25.09)	319 (35.80)
Gender, N (%)			
Male	898 (46.12)	7 (0.66)	891 (100.00)
Female	1049 (53.88)	1049 (99.34)	‐
Race, N (%)			
White	1115 (57.27)	638 (60.42)	477 (53.54)
African–American	592 (30.41)	288 (27.27)	304 (34.12)
Other/Unknown	240 (12.32)	130 (12.31)	110 (12.34)
Region^a^, N (%)			
Large Metro	1136 (58.35)	568 (53.79)	568 (63.75)
Small Metro/Non‐metro	811 (41.65)	488 (46.21)	323 (36.25)
Screening, N (%)			
Colonoscopy	998 (51.26)	467 (44.22)	482 (54.1)
No	949 (48.74)	589 (55.78)	409 (45.9)
BMI, mean (SD)	29.57 (6.19)	30.19 (6.97)	28.84 (5.05)
Hypertension, N (%)			
Yes	1427 (73.29)	691 (65.44)	736 (82.60)
No	520 (26.71)	365 (34.56)	155 (17.40)
Diabetes, N (%)			
Yes	544 (27.94)	250 (23.67)	294 (33.0)
No	1403 (72.06)	806 (76.33)	597 (67.0)

^a^ Large metropolitan areas defined as having a population of ≥250,000. Small metro areas have a population of <250,000.

Overall 51% of survivors had undergone a colonoscopy based on electronic health record data. However, there was a difference in CRC screening rate between prostate (54%) and breast (44%) cancer survivors. In logistic regression models adjusted for age, gender, race, region, and comorbidities (BMI, diabetes, and hypertension) in the overall survivorship sample, older age (≥65 years), being a breast cancer survivor compared to prostate cancer, and NHW race compared to NHB were associated with a lower probability of receiving CRC screening (Table 2). In multivariable adjusted models, survivors living in small metro areas were 3.62 times as likely to receive CRC screening compared to those in large metro areas (95% CI: 2.93, 4.47). Although BMI and diabetes were not associated with the receipt of CRC screening, survivors with hypertension were more than two times as likely to have received colonoscopy than those without hypertension (OR: 2.12, 95% CI: 1.67, 2.69); and the association was stronger for breast than prostate cancer patients. These findings remained statistically significant in stratified analyses by cancer type (breast and prostate) with some differences. Breast cancer survivors in small metro areas were five times as likely to get CRC screening compared to those in large metro areas (95% CI: 3.90, 7.05). This association was less strong among prostate cancer survivors (OR: 2.36, 95% CI: 1.20, 2.54).

**TABLE 2:** Association of sociodemographic characteristics, area of residence, and comorbid conditions with receipt of CRC screening in breast and prostate cancer survivors.

Characteristics	All survivors (N = 1,947)			Breast cancer survivors (N = 1,056)			Prostate cancer survivors (N = 891)		
	CRC screening, N (%)	Crude Odds ratio (95% CI)^b^	Adjusted Odds ratio (95% CI)^c^	CRC screening, N (%)	Crude Odds ratio (95% CI) ^b^	Adjusted Odds ratio (95% CI)^c^	CRC screening, N (%)	Crude Odds ratio (95% CI) ^b^	Adjusted Odds ratio (95% CI)^c^
Age at diagnosis									
<65	333 (48.76)	1.0	1.0	195 (43.24)	1.0	1.0	138 (59.48)	1.0	1.0
>=65	560 (44.3)	0.84 (0.69, 1.01)	0.7 (0.57, 0.86)	239 (39.5)	0.86 (0.67, 1.1)	0.76 (0.57, 1)	321 (48.71)	0.65 (0.48, 0.88)	0.62 (0.45, 0.85)
Gender									
Male	461 (51.34)	1.0	1.0	2 (28.57)	‐	‐	459 (51.52)	‐	‐
Female	432 (41.18)	0.66 (0.55, 0.79)	0.6 (0.49, 0.73)	432 (41.18)	‐	‐	‐	‐	‐
Race									
White	543 (48.7)	1.0	1.0	291 (45.61)	1.0	1.0	252 (52.83)	1.0	1.0
African–American	278 (46.96)	0.93 (0.76, 1.14)	1.29 (1.02, 1.62)	112 (38.89)	0.76 (0.57, 1.01)	1.25 (0.88, 1.75)	166 (54.61)	1.07 (0.8, 1.43)	1.33 (0.97, 1.82)
Region^a^									
Large metro	392 (34.51)	1.0	1.0	139 (24.47)	1.0	1.0	253 (44.54)	1.0	1.0
Small metro/Non‐metro	501 (61.78)	3.07 (2.54, 3.7)	3.62 (2.93, 4.47)	295 (60.45)	4.72 (3.62, 6.14)	5.25 (3.9, 7.05)	206 (63.78)	1.24 (0.93, 1.65)	2.36 (1.74, 3.2)
BMI									
<30	578 (45.84)	1.0	1.0	268 (41.88)	1.0	1.0	310 (49.92)	1.0	1.0
>=30	315 (45.92)	1 (0.83, 1.21)	0.96 (0.79, 1.18)	166 (39.9)	0.92 (0.72, 1.19)	0.83 (0.62, 1.1)	149 (55.19)	0.92 (0.72, 1.19)	1.17 (0.87, 1.59)
Hypertension									
No	174 (33.46)	1.0	1.0	110 (30.14)	1.0	1.0	64 (41.29)	1.0	1.0
Yes	719 (50.39)	2.02 (1.64, 2.49)	2.12 (1.67, 2.69)	324 (46.89)	2.05 (1.56, 2.68)	2.39 (1.74, 3.29)	395 (53.67)	1.65 (1.16, 2.34)	1.74 (1.2, 2.54)
Diabetes									
No	614 (43.76)	1.0	1.0	313 (38.83)	1.0	1.0	301 (50.42)	1.0	1.0
Yes	279 (51.29)	1.35 (1.11, 1.65)	1.05 (0.84, 1.31)	121 (48.4)	1.48 (1.11, 1.97)	1.13 (0.81, 1.57)	158 (53.74)	1.14 (0.86, 1.51)	1.02 (0.75, 1.37)

^a^ Large metropolitan areas defined as having a population of ≥250,000. Small metro areas have a population of <250,000.

^b^ Odds ratios comparing the odds of being current on CRC screening guidelines to no screening or not concordant with screening guidelines‐based logistic regression models.

^c^ Logistic regression models adjusted for age at diagnosis, gender, race, region, BMI, hypertension, and diabetes. Prostate cancer and breast cancer specific analyses do not adjust for gender.

In stratified analyses by residential status (Table 3), NHB prostate cancer survivors in large metro areas were 48% more likely to have CRC screening compared to NHW (95% CI: 1.03, 2.13). In contrast, race was not associated with CRC screening among prostate cancer survivors in small metro areas. Both breast and prostate cancer survivors with hypertension were more than two times as likely to have received CRC screening compared to those without hypertension in both large (95% CI: 1.55, 3.10) and small (95% CI: 1.50, 2.96) metro areas.

**TABLE 3:** Association of sociodemographic characteristics, area of residence, and comorbid conditions with receipt of CRC screening in breast and prostate cancer survivors by region ^a^.

Characteristics	Large Metro				Small Metro / Non‐metro			
	CRC screening, N (%)	All survivors (N = 1136)	Breast cancer survivors (N = 568)	Prostate cancer survivors (N = 568)	CRC screening, N (%)	All survivors (N = 811)	Breast cancer survivors (N = 488)	Prostate cancer survivors (N = 323)
		Adjusted Odds ratio (95% CI)^b^	Adjusted Odds ratio (95% CI)^b^	Adjusted Odds ratio (95% CI)^b^		Adjusted Odds ratio (95% CI)^b^	Adjusted Odds ratio (95% CI)^b^	Adjusted Odds ratio (95% CI)^b^
Age at diagnosis (in years)								
<65	131 (35.5)	1.0	1.0	1.0	202 (64.33)	1.0	1.0	1.0
>=65	261 (34.03)	0.71 (0.54, 0.95)	0.81 (0.54, 1.22)	0.62 (0.42, 0.93)	299 (60.16)	0.68 (0.5, 0.93)	0.71 (0.48, 1.06)	0.58 (0.34, 1)
Gender								
Male	254 (44.41)	1.0	‐	‐	207 (63.5)	1.0	‐	‐
Female	138 (24.47)	0.43 (0.33, 0.56)	‐	‐	294 (60.62)	0.94 (0.7, 1.28)	‐	‐
Race								
White	157 (31.91)	1.0	1.0	1.0	386 (61.96)	1.0	1.0	1.0
African–American	200 (42.19)	1.4 (1.06, 1.84)	1.31 (0.86, 2.01)	1.48 (1.03, 2.13)	78 (66.1)	1 (0.65, 1.54)	1.16 (0.63, 2.11)	0.82 (0.44, 1.53)
BMI								
<30	247 (34.12)	1.0	1.0	1.0	331 (61.64)	1.0	1.0	1.0
>=30	145 (35.19)	1.03 (0.78, 1.36)	0.76 (0.5, 1.14)	1.37 (0.93, 2)	170 (62.04)	0.9 (0.66, 1.23)	0.89 (0.6, 1.33)	0.92 (0.55, 1.53)
Hypertension								
No	56 (19.65)	1.0	1.0	1.0	118 (50.21)	1.0	1.0	1.0
Yes	336 (39.48)	2.2 (1.55, 3.1)	2.35 (1.45, 3.82)	2.00 (1.21, 3.29)	383 (66.49)	2.11 (1.5, 2.96)	2.15 (1.53, 3.03)	2.46 (1.61, 3.77)
Diabetes								
No	264 (32.04)	1.0	1.0	1.0	350 (60.45)	1.0	1.0	1.0
Yes	128 (41.03)	1.13 (0.84, 1.51)	1.09 (0.69, 1.74)	1.17 (0.81, 1.71)	151 (65.09)	0.99 (0.71, 1.4)	1.17 (0.72, 1.88)	0.87 (0.53, 1.43)

^a^ Large metropolitan areas defined as having a population of ≥250,000. Small metro areas have a population of <250,000.

^b^ Odds ratios comparing the odds of being current on CRC screening guidelines to no screening or not concordant with screening guidelines‐based logistic regression models. Adjusted for age at diagnosis, gender, race, region, BMI, hypertension, and diabetes. Prostate cancer and breast cancer specific analyses do not adjust for gender.

Similar results were obtained when we repeated our analyses only among those below the age of 75 years with the following exceptions. Age and race were no longer associated with CRC screening when the analytic dataset excluded those above the age of 75 years (Table S1). In addition, race was not associated with CRC screening in either large or small metro areas (Table S2).

## 4 DISCUSSION

To the best of our knowledge this is the first study to compare men and women cancer survivors, prostate and breast cancer survivors, respectively, with the receipt of medicalrecord verified CRC screening in a single health‐care system. In our analyses, prostate cancer survivors were more likely to undergo CRC screening than breast cancer survivors after adjusting for demographic, residential, and comorbidity factors. In addition, survivors living in small metro areas were more than three times as likely to receive CRC screening compared to those in large metro areas. Finally, although diabetes and overweight/obesity were not associated with increased likelihood of screening among cancer survivors, survivors with hypertension were more than two times as likely to be screened as those without hypertension. Although comorbid conditions are not recognized to be strong mediators of being current on CRC screening guidelines in the general population,^22^ our results suggest they might be a more important factor among cancer survivors. This could be related to the higher prevalence of hypertension in cancer survivors; and to the increased risk of hypertension associated with cancer therapies, such as chemotherapy, radiotherapy, and androgen‐deprivation therapy.^23^, ^24^, ^25^ This might potentially lead to increased primary care provider visits leading to better adherence with other health recommendations including CRC screening. However, we did not have data on primary care provider visits to evaluate this hypothesis.

Data on CRC screening uptake between different cancer survivor populations are sparse. Given existing disparities in CRC screening access and uptake it is important to determine whether such disparities are evident within the cancer survivor population. Although race‐based disparities between Black and White patients was not evident in our study, we did find gender disparities with prostate cancer survivors more likely to have received a colonoscopy within 10 years than breast cancer survivors. We did not find data on gender disparities in preventive care in survivorship in the literature. An analysis of the cancer survivorship care from the military health system for TRIACARE suggested that breast cancer patients were more likely to receive recommended preventive survivorship care compared to prostate and CRC survivors; and that there were geographic disparities in the quality of survivorship care.^26^ However, data on CRC screening were not compared between survivors of different cancers. Homan et al. conducted a study comparing CRC screening rates between breast cancer survivor and female survivors of other cancers using 2010 Behavioral Risk Factor Surveillance System (BRFSS).^9^ Compared to 75.4% of breast cancer survivors, female survivors of other cancers were less likely (70.8%) to be up to date on their colonoscopy screening but the difference was not statistically significant.^9^ This study was based on self‐reported screening data with a potential for biased recall; and did not compare prostate with breast cancer patients.

Results from our study suggest lower levels of colonoscopy screening (51.5%) in a community‐based practice in Southern Maryland compared to CRC screening levels based on nationally representative samples.^8^, ^9^, ^27^ In a sample of cancer survivors from the 2014 BRFSS, Shay et al. reported 81% of survivors being up‐to‐date with CRC screening.^27^ A similar finding was reported by Homan et al. using 2010 BRFSS data––compared to 75% of breast cancer survivors reporting a colonoscopy screening in the last 10 years only 60% of non‐cancer controls reported the same.^9^ Another study using data from the 2013 National Health Interview Survey (NHIS) reported a CRC screening rate of 63% among cancer survivors compared to 50% among age, sex, race/ethnicity, and geographic region matched cancer‐free controls from the same survey.^8^ There could be multiple reasons for the lower rates of CRC screening we observed in our population compared to nationally representative samples. It is possible that overall rates of CRC screening in Maryland are lower than the United States; and this is reflected among the cancer survivors. However, rates of CRC screening in Maryland are reported to be 70% in the 2016 BRFSS survey data.^28^ It is also possible that self‐reported rates from national samples overestimate CRC screening rates as a result of inaccurate self‐report or non‐response bias.

Screening rates for CRC in our study of cancer survivors (51.5%) are lower than those reported for the U.S. population (68.8%) in 2018.^29^ This is significantly short of the Healthy People 2020 target of 71%.^30^ Nationally, based on data from the 2018 Behavioral Risk Factor Surveillance System (BRFSS), women (70.5%) are more likely to be screened than men (67%).^29^ However, among cancer survivors we observed a higher rate of colonoscopy screening among men (54%) than women (44%). Similar to BRFSS data in the U.S. population, survivors aged 65 years or above in our study were more likely to be screened than younger survivors but age‐specific screening rates among survivors was lower than the general population. Our observations among survivors did not suggest CRC screening‐based Black and White disparities, which is also reflected in the BRFSS data; although rates for both Whites and Blacks in our study were much lower than that reported nationally.^29^ Another difference between our results and those reported in BRFSS 2018 is the higher rate of CRC screening seen in small metropolitan and non‐metropolitan areas compared to the larger metropolitan areas. In southern Maryland where this study was conducted, there are numerous satellite health‐care sites which increases the opportunities for CRC screening. This may explain the increased uptake of CRC screening in smaller areas, although not among those deemed rural. However, this difference should be interpreted with caution because the national data compared metropolitan to non‐metropolitan areas; and we did not have adequate representation of rural residents in our single‐system study.

Our analyses from a single, community‐based health‐care system had several strengths. A large sample size for prostate and breast cancer survivors ensured adequate power to determine gender disparities in colonoscopy screening in this population. CRC screening status and date of screening were based on medical record review and were not affected by potential recall issues associated with self‐reported screening. Health‐care system and insurance‐related factors were less likely to be large confounders in our analyses given that the data were collected from a single system with insured patients. Our study had several weaknesses. Although screening guidelines for CRC recommend tests other than colonoscopy for CRC screening, such as fecal immunochemical tests, we did not abstract data on these modalities and could have underestimated CRC screening in this population. We also did not have data to distinguish patients at average‐risk of CRC from those at high CRC risk who would be recommended colonoscopies in less than 10‐year intervals. This might have led us to over‐estimate CRC screening adherence in our study. We did not have data on non‐cancer controls and, therefore, cannot make direct conclusions on the rate of CRC screening among cancer survivors compared to the general population. In addition, given the differences in age and other demographic characteristics between cancer survivors from Southern Maryland in our study and the general non‐cancer U.S. population we cannot extrapolate our results to cancer survivors in other parts of the United States or Maryland. We also did not have an adequate sample size to determine race/ethnicity disparities beyond Non‐Hispanic Black‐White comparisons. Similarly, few survivors were from rural areas and we could not compare urban versus rural disparities in this sample. Finally, it is well known that the behaviors related to screening among cancer survivors is driven primarily by the health‐care provider, that is, oncologists or primary care physicians. However, we are unable to determine whether the survivors were recommended for CRC screenings or other preventive health services or if they are non‐adherent, based on the study methodology.

## 5 CONCLUSION

CRC screening rates remain low among breast and prostate cancer survivors with existing disparities observed based on gender and geographic area of residence. In addition, certain comorbidities are associated with increased adherence to colonoscopy screening and future studies should examine the mechanisms associated with these findings. Our findings suggest the importance of secondary cancer prevention in survivorship care plans for breast and prostate cancer patients and effective implementation of such plans within the primary care system.

## CONFLICT OF INTEREST

All the authors have nothing to disclose and have no conflict of interest.

## Supporting information

Table S1‐2

## Data Availability

The data that support the findings of this study are available from the corresponding author upon reasonable request.
