# Supplementary material for: Disparities in colorectal cancer screening among breast and prostate cancer survivors
Source: Cancer Med. 2021 Feb 5;10(4):1448–56. doi: 10.1002/cam4.3729 (PMC7926020; doi:10.1002/cam4.3729)
Supplement: Supplementary file 1 — Table S1‐2 [file CAM4-10-1448-s001.docx]

**APPENDIX: Supplementary Tables**

**Colorectal Cancer Screening Among Breast and Prostate Cancer Survivors in Southern Maryland**

Supplementary Table 1. Association of sociodemographic characteristics, area of residence, and comorbid conditions with receipt of CRC screening in breast and prostate cancer survivors (excluding survivors 75 years of age or above)

| Characteristics | All survivors  (N=1,363) | | | Breast cancer survivors  (N=791) | | | Prostate cancer survivors  (N=572) | | |
| --- | --- | --- | --- | --- | --- | --- | --- | --- | --- |
|  | CRC screening, N (%) | Crude Odds ratio (95% CI) ^2^ | Adjusted Odds ratio (95% CI) ^3^ | CRC screening, N (%) | Crude Odds ratio (95% CI) ^2^ | Adjusted Odds ratio (95% CI) ^3^ | CRC screening, N (%) | Crude Odds ratio (95% CI) ^2^ | Adjusted Odds ratio (95% CI) ^3^ |
| Age at diagnosis |  |  |  |  |  |  |  |  |  |
| <65 | 333 (48.76) | 1.0 | 1.0 | 195 (43.24) | 1.0 | 1.0 | 138 (59.48) | 1.0 | 1.0 |
| >=65 | 330 (48.53) | 0.99 (0.8, 1.23) | 0.83 (0.66, 1.05) | 143 (42.06) | 0.95 (0.72, 1.27) | 0.8 (0.58, 1.11) | 187 (55) | 0.83 (0.59, 1.17) | 0.84 (0.59, 1.2) |
| Gender |  |  |  |  |  |  |  |  |  |
| Male | 326 (56.6) | 1.0 | 1.0 | 1 (25) | - | - | 325 (56.82) | - | - |
| Female | 337 (42.82) | 0.57 (0.46, 0.71) | 0.5 (0.39, 0.64) | 337 (42.82) | - | - | - | - | - |
| Race |  |  |  |  |  |  |  |  |  |
| White | 395 (51.5) | 1.0 | 1.0 | 221 (46.82) | 1.0 | 1.0 | 174 (58.98) | 1.0 | 1.0 |
| African American | 206 (48.7) | 0.89 (0.7, 1.13) | 1.23 (0.93, 1.62) | 92 (41.82) | 0.82 (0.59, 1.13) | 1.28 (0.86, 1.9) | 114 (56.16) | 0.89 (0.62, 1.28) | 1.17 (0.78, 1.74) |
| Region^1^ |  |  |  |  |  |  |  |  |  |
| Large Metro≥250,000 | 277 (36.3) | 1.0 | 1.0 | 103 (25.56) | 1.0 | 1.0 | 174 (48.33) | 1.0 | 1.0 |
| Small Metro<250,000/Nonmetro | 386 (64.33) | 3.16 (2.53, 3.96) | 4.00 (3.1, 5.16) | 235 (60.57) | 4.47 (3.3, 6.06) | 5.13 (3.65, 7.22) | 151 (71.23) | 1.08 (0.76, 1.53) | 2.86 (1.94, 4.23) |
| BMI**** |  |  |  |  |  |  |  |  |  |
| <30 | 404 (49.03) | 1.0 | 1.0 | 198 (43.33) | 1.0 | 1.0 | 206 (56.13) | 1.0 | 1.0 |
| >=30 | 259 (48.05) | 0.96 (0.77, 1.2) | 0.97 (0.76, 1.23) | 140 (41.92) | 0.94 (0.71, 1.26) | 0.87 (0.63, 1.2) | 119 (58.05) | 0.94 (0.71, 1.26) | 1.11 (0.77, 1.6) |
| Hypertension |  |  |  |  |  |  |  |  |  |
| No | 151 (36.47) | 1.0 | 1.0 | 97 (32.33) | 1.0 | 1.0 | 54 (47.37) | 1.0 | 1.0 |
| Yes | 512 (53.95) | 2.04 (1.61, 2.59) | 2.13 (1.62, 2.8) | 241 (49.08) | 2.02 (1.49, 2.72) | 2.33 (1.63, 3.32) | 271 (59.17) | 1.61 (1.07, 2.43) | 1.82 (1.16, 2.83) |
| Diabetes |  |  |  |  |  |  |  |  |  |
| No | 472 (47.2) | 1.0 | 1.0 | 249 (40.82) | 1.0 | 1.0 | 223 (57.18) | 1.0 | 1.0 |
| Yes | 191 (52.62) | 1.24 (0.98, 1.58) | 0.96 (0.73, 1.25) | 89 (49.17) | 1.4 (1, 1.96) | 1.09 (0.74, 1.61) | 102 (56.04) | 0.95 (0.67, 1.36) | 0.84 (0.57, 1.22) |
| ^1^ Large metropolitan area defined as having a population of ≥250,000. Small metro areas have a population <250,000.  ^2^ Odds ratios comparing the odds of being current on CRC screening guidelines to no screening or not concordant with screening guidelines based on logistic regression models.  ^3^Logistic regression models adjusted for age at diagnosis, gender, race, region, BMI, hypertension and diabetes. Prostate- and breast-cancer specific analyses do not adjust for gender. | | | | | | | | | |

Supplementary Table 2. Association of sociodemographic characteristics, area of residence, and comorbid conditions with receipt of CRC screening in breast and prostate cancer survivors by region^1^

| Characteristics | Large Metro≥250,000 | | | | Small Metro<250,000/Nonmetro | | | |
| --- | --- | --- | --- | --- | --- | --- | --- | --- |
|  | CRC screening, N (%) | All survivors | Breast cancer survivors | Prostate cancer survivors | CRC screening, N (%) | All survivors | Breast cancer survivors | Prostate cancer survivors |
|  |  | Adjusted Odds ratio (95% CI)^2^ | Adjusted Odds ratio (95% CI)^2^ | Adjusted Odds ratio (95% CI)^2^ |  | Adjusted Odds ratio (95% CI)^2^ | Adjusted Odds ratio (95% CI)^2^ | Adjusted Odds ratio (95% CI)^2^ |
| Age at diagnosis (in years) |  |  |  |  |  |  |  |  |
| <65 | 131 (35.5) | 1.0 | 1.0 | 1.0 | 202 (64.33) | 1.0 | 1.0 | 1.0 |
| 65-74 | 146 (37.06) | 0.84 (0.61, 1.15) | 0.9 (0.56, 1.44) | 0.78 (0.5, 1.21) | 184 (64.34) | 0.82 (0.58, 1.17) | 0.73 (0.47, 1.14) | 0.91 (0.49, 1.69) |
| Gender |  |  |  |  |  |  |  |  |
| Male | 174 (48.07) | 1.0 | - | - | 152 (71.03) | 1.0 | - | - |
| Female | 103 (25.69) | 0.41 (0.3, 0.56) | - | - | 234 (60.62) | 0.68 (0.47, 0.99) | - | - |
| Race |  |  |  |  |  |  |  |  |
| White | 104 (33.12) | 1.0 | 1.0 | 1.0 | 291 (64.24) | 1.0 | 1.0 | 1.0 |
| African American | 143 (43.2) | 1.32 (0.94, 1.86) | 1.41 (0.85, 2.35) | 1.22 (0.77, 1.95) | 63 (68.48) | 0.99 (0.6, 1.63) | 1.17 (0.6, 2.26) | 0.74 (0.34, 1.59) |
| BMI |  |  |  |  |  |  |  |  |
| <30 | 155 (35.07) | 1.0 | 1.0 | 1.0 | 249 (65.18) | 1.0 | 1.0 | 1.0 |
| >=30 | 122 (38.01) | 1.11 (0.8, 1.53) | 0.84 (0.52, 1.35) | 1.39 (0.89, 2.17) | 137 (62.84) | 0.81 (0.57, 1.17) | 0.89 (0.57, 1.38) | 0.7 (0.36, 1.35) |
| Hypertension |  |  |  |  |  |  |  |  |
| No | 43 (20.19) | 1.0 | 1.0 | 1.0 | 108 (53.73) | 1.0 | 1.0 | 1.0 |
| Yes | 234 (42.55) | 2.45 (1.64, 3.67) | 2.42 (1.38, 4.22) | 2.37 (1.31, 4.3) | 278 (69.67) | 1.91 (1.3, 2.81) | 2.35 (1.46, 3.76) | 1.27 (0.61, 2.65) |
| Diabetes |  |  |  |  |  |  |  |  |
| No | 193 (34.84) | 1.0 | 1.0 | 1.0 | 279 (62.56) | 1.0 | 1.0 | 1.0 |
| Yes | 84 (40.19) | 0.92 (0.64, 1.31) | 1.13 (0.66, 1.95) | 0.77 (0.48, 1.23) | 107 (69.48) | 1.05 (0.69, 1.6) | 1.04 (0.59, 1.82) | 1.12 (0.58, 2.17) |
| ^1^ Large metropolitan area defined as having a population of ≥250,000. Small metro areas have a population <250,000.  ^2^ Odds ratios comparing the odds of being current on CRC screening guidelines to no screening or not concordant with screening guidelines based on logistic regression models. Adjusted for age at diagnosis, gender, race, region, BMI, hypertension and diabetes. Prostate- and breast-cancer specific analyses do not adjust for gender. | | | | | | | | |
